# The Influence of Temperature on the Hydration Rate of Cements Based on Calorimetric Measurements

**DOI:** 10.3390/ma14113025

**Published:** 2021-06-02

**Authors:** Włodzimierz Kiernożycki, Jarosław Błyszko

**Affiliations:** Department of Reinforced Concrete Structures and Concrete Technology, Faculty of Civil and Environmental Engineering, West Pomeranian University of Technology in Szczecin, Aleja Piastów 50a, 70-311 Szczecin, Poland; Wlodzimierz.Kiernozycki@zut.edu.pl

**Keywords:** cement, isothermal calorimetry, heat of hydration, concrete maturity

## Abstract

The study presents results of calorimetric tests of three different cements. Two Ordinary Portland cements, CEM I 52.5 R and CEM I 42.5 R, and one Blastfurnace cement, CEM III/A 42.5 N LH/HSR/NA, were analysed. The analysis has shown that the empirical formulas derived based on the results can successfully replace the Arrhenius formula in determination of the hydration rate in relation to curing temperature. It was proven that the hydration rate in relation to the curing temperature changes with the progression of hydration. The study introduces an *E_n_* coefficient which determines the influence of curing temperature on generation of heat. Results of the study have shown that the value of *E_n_* is not constant and changes with the progression of hydration process. Proposed method of numerical modelling of the total heat generated and generation rate based on obtained results allows for the calculation of those two parameters for any curing conditions.

## 1. Introduction

Design of concrete constructions requires not only including the loads occurring during their service life but also the ones that can appear during the execution stage. Indirect loads generated by the hydration of cement-based materials and direct loads from execution processes are both present. Analysis of the after-effects of the indirect (thermal and shrinkage) and direct loads (dead loads) requires determining the influence of temperature on the hydration processes of concrete [1,2].

The heat generated by the hydration process during execution of concrete is the major cause of uneven heat distribution in massive elements [3]. Heat distribution and time of temperature equalization is influenced by different heat generation rates and total amount of generated heat. Thus, it is necessary to use admixtures for control of generated heat and to conduct tests to determine the heat generation rates of concrete. Based on initial test results, certain preventive actions are taken, including using low-heat cements, increasing aggregate content in the mix, conducting measurements of the temperature during execution or cooling with water [4]. In recent years, due to dynamic development of admixtures and FEM modelling, the issue of heat generation was studied by various authors [5,6,7,8,9,10,11].

The review of existing studies has shown different ways to describe the influence of curing temperature on the hydration rate of cement and corresponding strength development [12,13,14,15,16,17]. A.G. Saul [18] has proposed a time-temperature factor (TTF), also known as a maturity index, as a way to express the development of concrete’s strength. Rastrup [19] has introduced an equivalent age concept based on the van’t Hoff’s chemical principle in which the rate of reaction doubles with the increase of temperature by 10 °C.

The issue of determining the equivalent maturing time in different temperatures was studied by many researchers [20,21,22,23,24,25]. Currently two procedures are used to determine the maturity of executed concrete in reference to standard curing temperature (20 °C). These procedures are Standard Practice for Measuring Hydration Kinetics of Hydraulic Cementitious Mixtures Using Isothermal Calorimetry (ASTM C1679-08) [26] and Standard Practice for Estimating Concrete Strength by the Maturity Method (ASTM C1074_11) [27].

Both methods in their principle refer to Arrhenius equation, which, as opposed to both above mentioned propositions allows to include also the properties of used cement. The reserved attitude towards this approach results from use of the activation energy E. Kurdowski and Pichniarczyk [28] have objected to using the energy activation, that comes from the kinetic gas theory, for considerations of cement hydration.

Despite numerous studies [23,24,29,30,31,32,33], there is still no consensus on which approach and variables should be used for the purpose of maturity method (i.e., apparent activation energy).

The study presents the results of calorimetric tests of different cements under various maturing conditions. Results of the study allowed for the proposal of a method for describing the influence of curing temperature on heat generation of studied cements. Empirical formulas that include the influence of physicochemical properties of cements for the studied range of temperatures were proposed. To correlate the results acquired for the reference temperature of 20 °C, the study introduces an *E_n_* coefficient for each of studied cements. The proposed approach showed good correlation of the results.

## 2. Materials and Methods

### 2.1. Materials

For the purpose of this study, three different types of cement were chosen: Ordinary Portland Cement CEM I 52.5 R, CEM I 42.5 R and Blastfurnace Cement CEM III/A 42.5 N- LH/HSR/NA (CEM III 42.5 N), all manufactured by Górażdże Cement in Chorula, Poland. Cement characteristics are given in Table 1.

### 2.2. Test Procedure

Heat of hydration and heat flow was determined in a three-channel isothermal calorimeter TAM AIR by TA Instruments (New Castle, DE, USA). The dual-channel system allows one to test simultaneously cement specimen and reference specimen. The software allows one to measure the heat in extended periods with a measuring error of ±0.02 °C. Test specimen and the equipment were prepared in accordance to EN 196-11 [34].

Cement paste specimen with a water–cement ratio of 0.5 was used in the study. After acquiring base temperature by the paste components and calorimeter, the specimens were prepared. Water (15 g) was added to cement (30 g) and mixed for 60 s by hand in a container used for calorimeter. The container was insulated with a cloth to block the heat coming from hand. The container was immediately placed into the calorimeter with base line prepared. Time between adding water to first measurement did not exceed 2 min. The reference specimen was prepared by replacing the cement with a silica sand. Directly after mixing, the samples were set on a 7-day long cycle where generated heat and heat flow were measured. The study was performed for different curing temperatures of 20 °C, 25 °C, 30 °C and 40 °C.

## 3. Results

**Figure 1:**
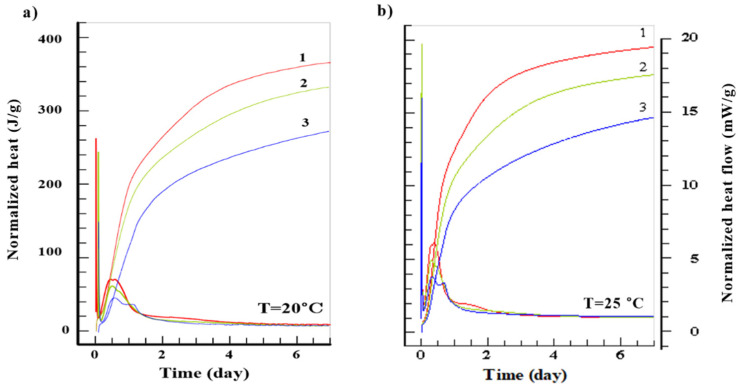
Amount of generated heat (J/g) and heat flow (mW/g) of studied cements: 1. CEM I 52.5 R, 2. CEM I 42.5 R, 3. CEM III 42.5 N for curing temperatures 20 °C (**a**) 25 °C (**b**).

**Figure 2:**
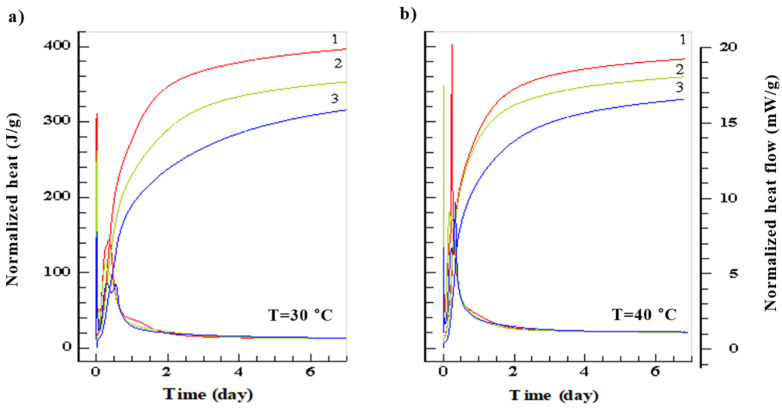
Amount of generated heat (J/g) and flow (mW/g) of studied cements: 1. CEM I 52.5 R, 2. CEM I 42.5 R, 3. CEM III 42.5 N for curing temperatures 30 °C (**a**) 40 °C (**b**).

Results of generated heat and heat flow for different cements are presented in Figure 1 and Figure 2 and Table 2.

After 7 days of maturing the highest normalized heat of hydration, regardless of the curing temperature, was generated by the CEM I 52.5 R (*Q*7 = 366 ÷ 396 J/g), and the lowest was generated by CEM III 42.5 N (*Q*7 = 272 ÷ 330 J/g). Heat flow was again the highest for CEM I 52.5 R (*dQ*/*dτ* = 3.06 ÷ 19.17 mW/g), while the lowest for CEM III 42.5 N (*dQ*/*dτ* = 1.93 ÷ 8.64 mW/g). With the increase of the curing temperature the maximum heat flow *dQ*/*dτ* also increases. However, this does not correspond to highest total heat generated throughout the whole cycle. Detailed results of heat flow and total heat generated are presented in Table 2.

Initial rapid increase in heat generation (first peak) is caused by the absorption of water by the cement grains and chemical reaction on their surface. The second peak is caused by the intensified formation of the C-S-H gel, AF_t_ phase and CH. Of importance is also production of the 3CaO·Al_2_O_3_ and 4CaO·3Al_2_O_3_·SO_4_ which limits the hydration of C_3_A [35]. With the increase of the surface area of cement and curing temperature, the heat generation rate also increases. The third peak clearly visible in case of the blastfurnace cement, especially for higher curing temperatures, is caused by the activation of the slag by the Ca(OH)_2_ and SO_4_^2−^ ions [36]. This additional peak, found sometimes in other cements at the end of the 4th stage of hydration [37], is caused by the hydration of remaining C_3_A and creation of hexagonal aluminates.

## 4. Discussion

Results presented in this study do not allow one to directly correlate the influence of curing temperature on the maturing of specimen. To determine the correlation, the following equation was introduced (1):

(1) $$v=A_{o}\exp\left(-\frac{E_{n}}{T}\right),$$

in which the reaction rate *v* is expressed as an exponential function of *E_n_* constant and curing temperature *T* in Celsius degrees. The constants *A_o_* and *E_n_* can be determined based on the experimental results of reaction rate *v* in different curing conditions. Equation (1) can be transformed to:

(2) $$lnv=lnA_{o}-\frac{E_{n}}{T}=B-\frac{E_{n}}{T}$$

where the temperature *T* is expressed in Celsius and *E_n_* determines the influence of temperature on heat generation. The *lnv* = *f*(1/*T*) graph of (2) shows linear function with a slope of tg∝ = *E_n_*.

**Figure 3:**
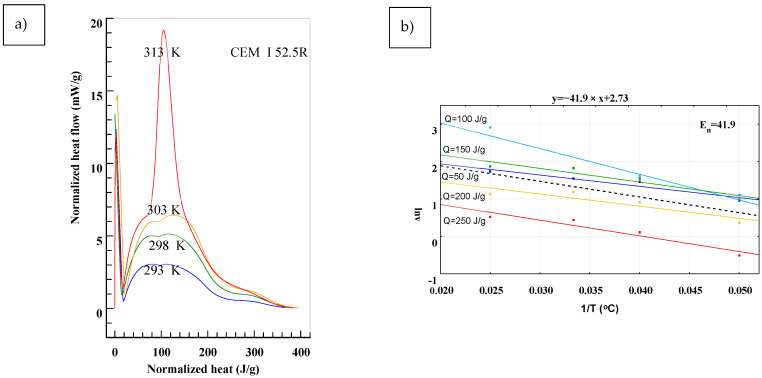
Determination of the *En* from the Equation (2) for CEM I 52.5 R. (**a**) Normalized heat flow in comparison to total heat generated for different temperatures, (**b**) Values of *En* for different temperatures and it’s representative value calculated using least-square method.

**Figure 4:**
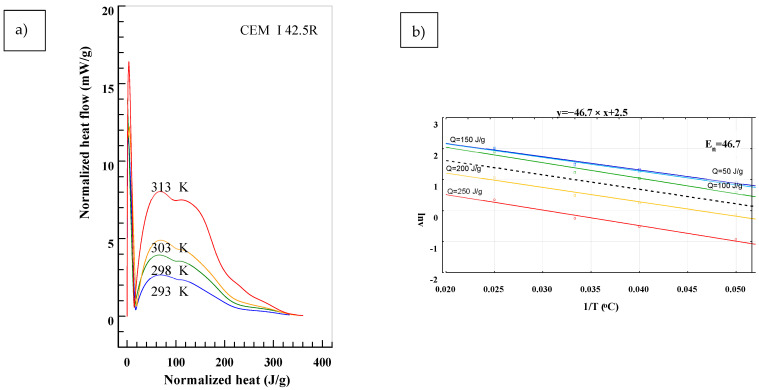
Determination of the *En* from the Equation (2) for CEM I 42.5 R. (**a**) Normalized heat flow in comparison to total heat generated for different temperatures, (**b**) Values of *En* for different temperatures and it’s representative value calculated using least-square method.

**Figure 5:**
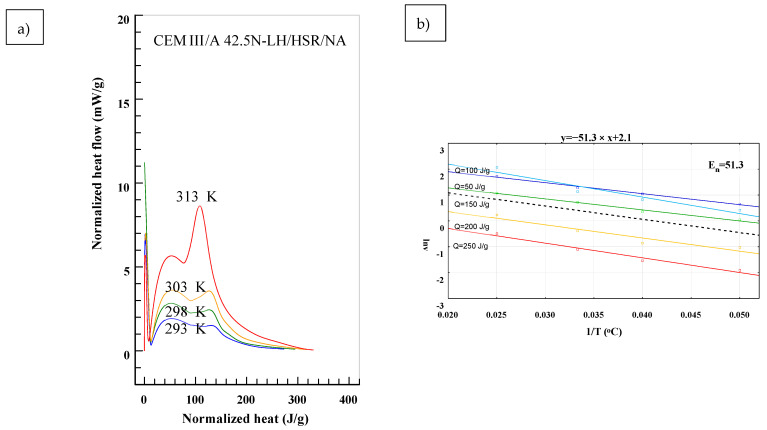
Determination of the *E_n_* from the Equation (2) CEM III 42.5 N. (**a**) Normalized heat flow in comparison to total heat generated for different temperatures, (**b**) Values of *En* for different temperatures and it’s representative value calculated using least-square method.

To determine the *E_n_* in accordance to (2), results presented in Figure 1 were transformed into:

(3) $$\frac{dQ\left(T\right)}{d\tau }=f\left(Q\right),$$

in which the *T* stands for different temperatures of maturing. Equation (3) determines the heat flow of cement under different curing temperatures at a given time as a total generated heat *Q*. The analysis for test specimen is presented in Figure 3, Figure 4 and Figure 5. The *E_n_* was determined for different levels of heat generated: *Q* = 50 J/g, *Q* = 100 J/g, *Q* = 150 J/g, *Q* = 200 J/g and *Q* = 250 J/g. Based on presented results, the slope of function (2) changes in time and depends on the hydration process stage. Biggest differences are observable in the first stage of hydration for the range of *Q* = 50 ÷ 150 J/g. In later stages the lines are almost parallel, meaning that the values of E_n_ are similar. In Figure 3, Figure 4 and Figure 5, the mean value from all of the measurements was marked with a black dotted line.

**Figure 6:**
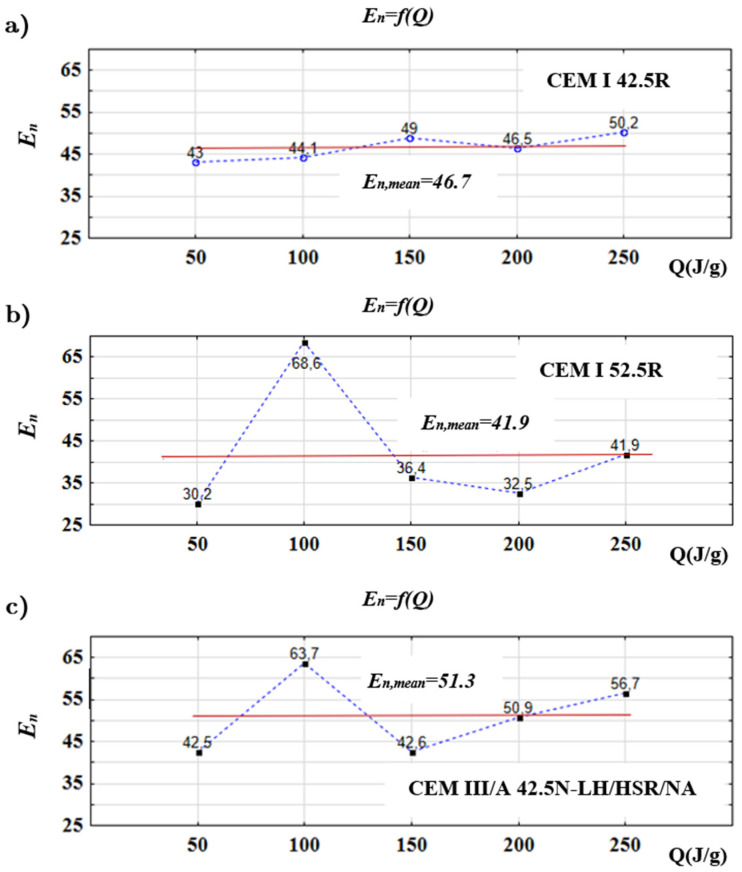
Values of the *E_n_* in relation to the total heat generated Q calculated for studied cements (**a**) CEM I 52.5 R, (**b**) CEM I 42.5 R, (**c**) CEM III A 42.5 N.

Figure 6 presents the values of the *E_n_* in relation to the level of total heat generated *Q*: 50, 100, 150, 200 and 250 J/g.

Results of the study have shown that the value of the *E_n_* is not constant. The value of *E_n_* changes with the progression of hydration process, which can be related to changes in the processes responsible for its rate. Performed tests have shown that after generating 100 J/g of heat, CEM I 52.5 R and CEM IIIA 42.5 N have higher values than the CEM I 42.5 R of the *E_n_* parameter (respectively *E_n_* = 68.8 and *E_n_* = 63.7 to *E_n_* = 44.1). In the opinion of the authors, this is probably caused by the higher surface area of two former cements equal to 4411 cm^2^/g and 4636 cm^2^/g compared to 3717 cm^2^/g for CEM I 42.5 R. It is worth mentioning that the maximum values of *E_n_* occur when the heat flux *dQ*/*dτ* is also the highest.

To determine the susceptibility of studied cements to changes in temperature during hydration, it is better to refer to the mean value of the *E_n_*. Results presented in Figure 5 allowed one to draw a conclusion that CEM I 52.5 R, with the highest strength, has the lowest value of *E_n_* = 41.9, meaning its susceptibility to temperature changes is the lowest.

The blastfurnace cement has the highest value of *E_n_* = 51.3 in this study, meaning it is the most susceptible to temperature changes from all studied cements. It was observed that the CEM I 42.5 with the highest surface area had the most linear *E_n_* = *f*(*Q*) function and *E_n_* = 46.7. The statement was rephrased. When comparing the results of conducted tests to data presented in Table 1, it can be noticed that the hydration heat and susceptibility to curing temperature is different between the cements. Analysis of the thermal stresses caused by the hydration heat in mass construction should be made taking into consideration detailed data on the hydration heat and heat flux for used cement.

**Figure 7:**
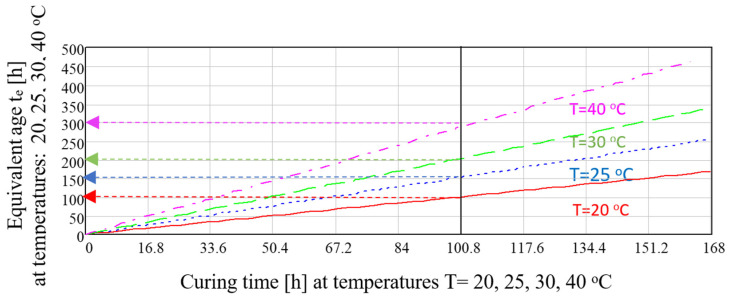
Equivalent age *t_e_* for CEM I 52.5 R (*E_n_* = 41.9) and different curing temperatures.

Figure 7 presents an example of temperature influence calculated based on (4) on the equivalent time *t_e_* of CEM I 52.5 R in different temperatures. Based on the results it can be said that the maturing time *t* = 100 h in *T* = 40 °C equals equivalent time *t_e_* = 300 h in *T_a_* = 20 °C.

By analysing the influence of temperature *T* on the amount of generated heat of hydration *Q*(*T*,*t_e_*) = *Q*(*T_a_*,*t*) based on (1), the equivalent time was derived *t_e_* = *f*(*t*):

(4) $$t_{e}=\exp\left[-\frac{E_{n}}{T}\left(\frac{T_{a}-T}{T_{a}}\right)\right]t$$

**Figure 8:**
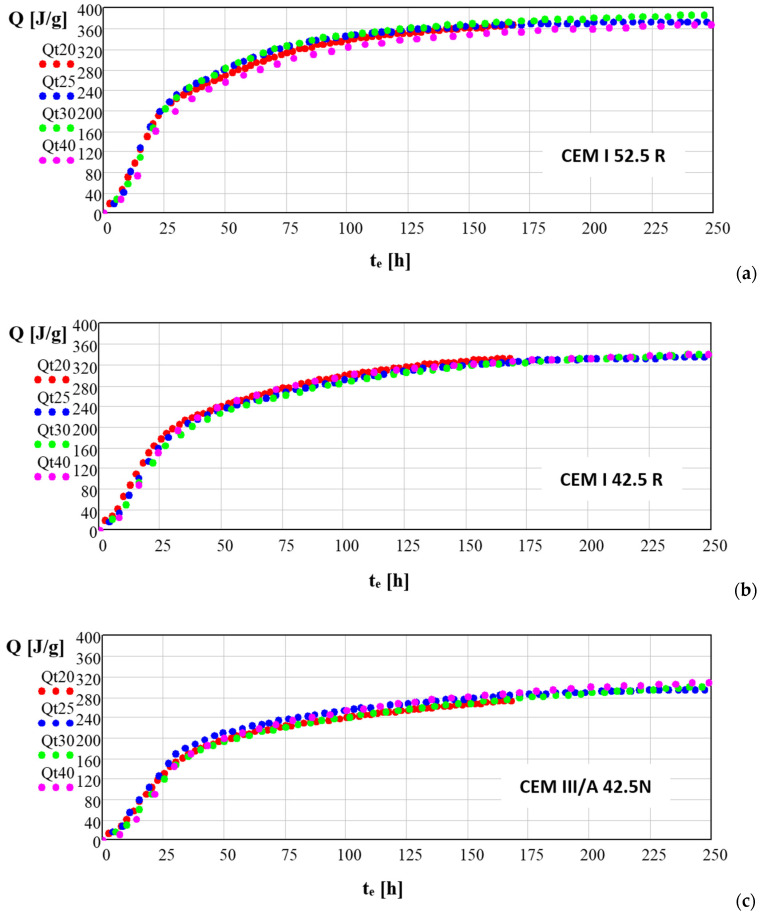
Generated heat *Q* (J/g) for studied cements in equivalent time function at curing temperatures of 20, 25, 30 and 40 °C. calculated for studied cements (**a**) CEM I 52.5 R, (**b**) CEM I 42.5 R, (**c**) CEM III A 42.5 N.

Figure 8 presents the heat generated by hydration of studied cements in *T* = 20, 25, 30 i 40 °C as a function of equivalent time *t_e_* calculated with (5). The equivalent time *t_e_* expressed by (5) and derived from (1) allows one to transform the results of the generated heat of hydration *Q* in reference temperature *T_a_* to expect values of generated heat in any given temperature *T*. Numerical modelling of the hydration processes has a great significance in analysing the indirect load caused by the heat of hydration kinetics, particularly in mass concretes.

Many experimental numerical models calculate the development of hydration in relation to its chemical composition, surface area, water–cement ratio, internal pressure or maturing temperature. Validation of those models is typically based on calorimetric measurements. As the number of characteristics influences the heat of hydration in cementitious materials, determination of which of them has the greatest influence requires extensive testing [38]. Several studies proposed simpler modelling methods with parameters assumed for particular cement types which were determined in experimental tests.

Among those studies presented in [39], the model proposed by Wesche [40] seems particularly interesting. The heat of hydration of different cements is calculated as:

(5) $$Q_{t}=Q\cdot \exp\left(P_{1}\cdot t^{P_{2}}\right),$$

where *Q* is the heat of hydration, *P*_1_ i *P*_2_ are the parameters related to class and type of cement and *t* is the time of hydration. The function (5) works in a range of $t\;\left(t\to 0^{+},\;\infty \right)$ assuming values between 0 ÷ *Q*. The derivative of the function (6):

(6) $$\frac{dQ}{dt}=W_{t}=Q_{t}\cdot P_{1}\cdot P_{2}\cdot t^{\left(P_{2}-1\right)},$$

assumes values of $W_{t}=0$ at $t\to 0^{+}$ and *t* = ∞, and reaches its peak also in this range. Wesche has estimated the mean values of the *P*_1_ and *P*_2_ for higher class cements (in accordance to DIN standards): Z 55 (*P*_1_ = −11.1 i *P*_2_ = −1.0) and Z 25 L (*P*_1_ = −74.8 i *P*_2_ = −1.5).

Further analysis of the kinetics of hydration processes of studied cements in various temperatures was made using transformed (5) in which the absolute hydration time *t* was replaced by the equivalent time *t_e_*, including the induction time *t_i_*.

The estimation of unknown parameters of Equation (5) for all three studied cements and reference temperatures allowed for the derivation of the following formulas:

For CEM I 52.5:

(7) $$Q_{o20\left(t\right)}=353\cdot \exp\left(-21.4\cdot {\left(t-1.5\right)}^{-0.94}\right)\;(t_{i}=1.5\;h\;E_{n}=41.9)$$

For CEM I 42.5 R:

(8) $$Q_{o20\left(t\right)}=345\cdot \exp\left(-16.0\cdot {\left(t-1.5\right)}^{-0.97}\right)\;(t_{i}=1.5\;h\;E_{n}=46.7)$$

For CEM III/A-42.5N:

(9) $$Q_{o20\left(t\right)}=305\cdot \exp\left(-9.7\cdot {\left(t-3.0\right)}^{-0.77}\right)\;(t_{i}=3.0\;h\;E_{n}=51.3)$$

**Figure 9:**
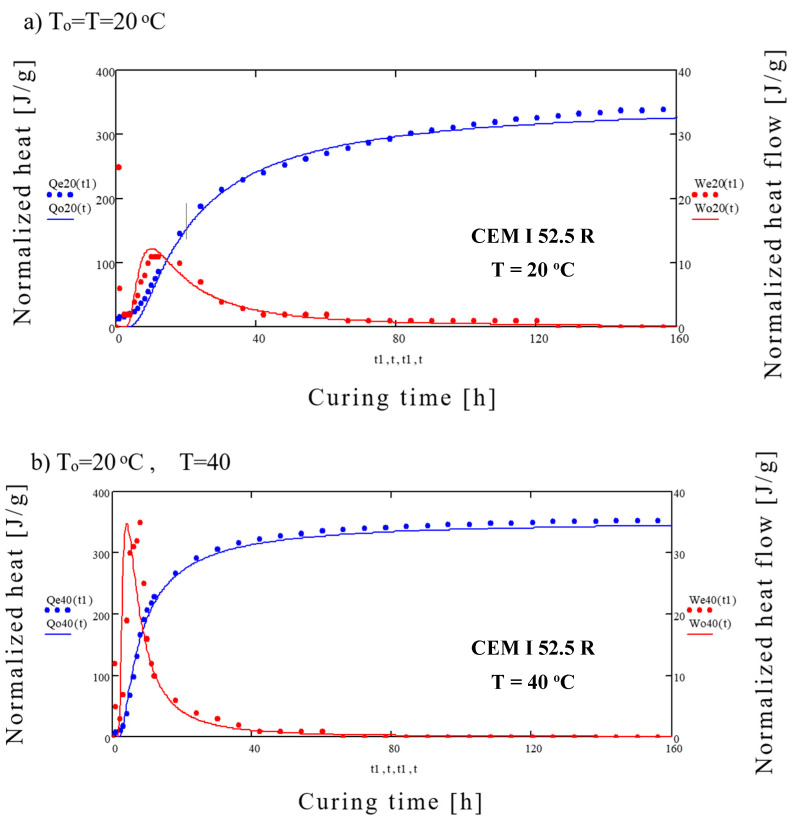
Comparison of experimental and calculated results of normalized heat and heat flow for the CEM I 52.5 R cement (**a**) for *T* = 20 °C, (**b**) for *T* = 40 °C.

Figure 9a presents an example of comparison between experimental results of heat generation *Q*_*e*20_ and results of *W*_*e*20_ calculated using (7) for CEM I 52.5 R and reference time *T*_*o*_ = *T* = 20 °C. Figure 9b shows the experimental results for temperature of 40 °C (*Q*_*e*40_ i *W*_*e*40_) in comparison to results modelled using (4) and *T_o_* = 20 °C and *T* = 40 °C.

**Figure 10:**
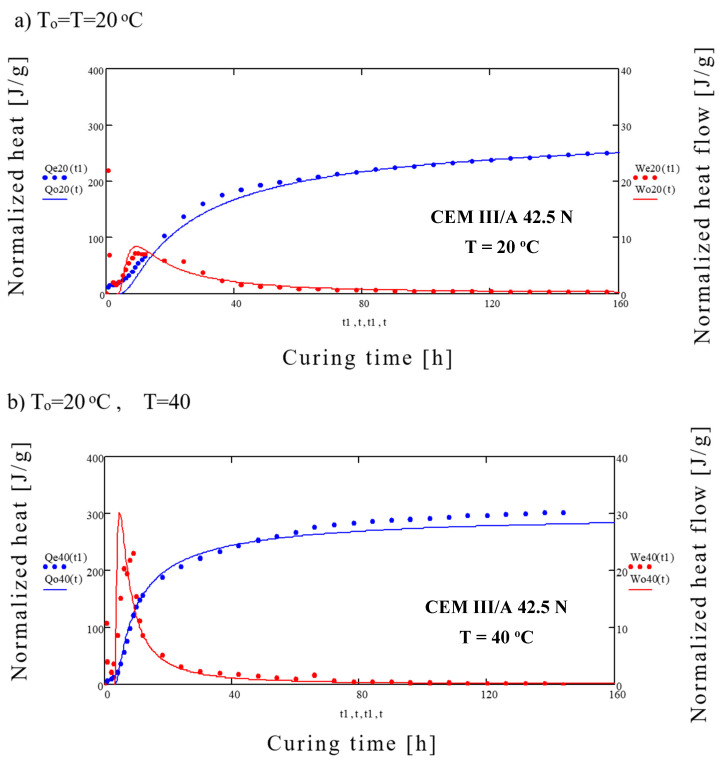
Comparison of experimental and calculated results of normalized heat and heat flow for the CEM III/A 42.5 N cement (**a**) for *T* = 20 °C, (**b**) for *T* = 40 °C.

Similar analysis was performed for blastfurnace slag, results of which are presented in Figure 10.

Relatively good compliance of the test results and calculated model was acquired in the study. Presented simple method of numerical modelling of generated heat of hydration and heat flow for determined influence of temperature *E_n_* allows for the transformation of the reference results to any given hydration conditions. The concept, however, requires further studies for different cement pastes and concretes cured in isometrical and adiabatic conditions.

## 5. Conclusions

The conducted tests performed on three different types of cements have shown that replacing classic Arrhenius formula with empirical equations that take into account the influence of curing temperature on hydration heat can provide good evaluation of susceptibility of those cements to temperature changes. Results of this study have shown that the susceptibility of cement to thermal conditions changes with the development of hydration process. Mean values of the susceptibility parameter determined for the first 7 days of hydration vary for different cement class and type. The presented simplified method of numerical modelling of heat generation and generation rate for determined value of the *E_n_* parameter (influence of curing temperature on cement hydration) allows for the recalculation of the results acquired in reference conditions for any given temperature.

## Figures and Tables

**Table 1:** Properties of cements used in the study.

Characteristic	CEM I 52.5 R	CEM I 42.5 R	CEM III 42.5 N
Composition [%]			
Portland clinker	95 ÷ 100	95 ÷ 100	35 ÷ 64
Ground granulated blast furnace slag	-	-	36 ÷ 65
Secondary components	0 ÷ 5	0 ÷ 5	0 ÷ 5
Compressive strength [MPa]			
2 days	36.2	29.0	14.8
28 days	63.6	56.9	58.3
Setting time (initial) [min]	170	184	201
Surface area (Blaine) [cm^2^/g]	4411	3717	4636
Chemical composition [%]			
SO_3_	2.93	2.93	2.70
Cl^−^	0.067	0.066	0.080
Loss on ignition	3.73	3.40	1.08
Insoluble residue	0.72	0.70	0.48
Heat of hydration (7 days) [J/g]	325 ÷ 375	325 ÷ 375	<270

The amount of gypsum in studied cements was <5% as required by European Standards.

**Table 2:** Results of calorimetric tests for studied cements.

Cement	Temperature (°C)	*Q*_7_^1^ (J/g)	max. dQ/dτ (mW/g)	${\tau_{\max}}^{3}$ (h, min)
CEM I 52.5 R	20	366	3.06	12 h 5 min
	25	390	5.10	8 h 50 min
	30	396	6.38	7 h 45 min
	40	384	19.17	5 h 50 min
CEM I 42.5 R	20	332	2.66	10 h 45 min
	25	352	3.92	7 h 50 min
	30	353	4.91	6 h 10 min
	40	360	7.90	4 h 10 min
CEM III 42.5 N	20	272	1.93	11 h 25 min
	25	294	2.77	8 h 55 min
	30	316	3.59	7 h 30 min
	40	330	8.64	8 h 15 min

^1^ *Q*_7_—Heat of hydration. ^2^ Maximum value of normalized heat. ^3^ Time in which *dQ*/*dτ* reaches maximum, h (hours), min (minutes).

## Data Availability

Data available on request due to file type and size.
